# Polarization-insensitive perfect absorption in van der waals hyper-structure

**DOI:** 10.1038/s41598-024-60891-0

**Published:** 2024-05-02

**Authors:** Muhammad Imran, Muhyiddeen Yahya Musa, Sajid Rauf, Dajiang Lu, Rujiang Li, Yibin Tian

**Affiliations:** 1 College of Mechatronics and Control Engineering, Shenzhen University, Shenzhen, 518000 China; 2 Department of Agriculture and Bio-Environmental Engineering Technology, Audu Bako College of Agriculture Dambatta, Kano, Nigeria; 3 National Key Laboratory of Radar Detection and Sensing, School of Electronic Engineering, Xidian University, Xi’an, 710071 China

**Keywords:** Infrared perfect absorption, Polarization, Graphene, Hexagonal Boron Nitride (hBN), Hyper-structure, Optical properties and devices, Nanophotonics and plasmonics, Metamaterials

## Abstract

Infrared perfect absorption has been widely investigated due to its potential applications in photodetectors, photovoltaics and medical diagnostics. In this report, we demonstrate that at particular infrared frequencies, a simple planar structure made up of graphene-hexagonal Boron Nitride (hBN) hyper-structure is able to nearly perfectly absorb incident light irrespective of its polarization (Transverse-Magnetic *TM,* or Transverse-Electric *TE*). By using this interferenceless technique, the hyper-structure achieves nearly zero reflectance at a wide range of angles in a narrow frequency band. We analytically predict the condition of achieving such an important feature of perfect absorption for both *TM* and *TE* polarizations. Interestingly, the infrared perfect absorption can be redshifted by increasing the thickness of the hBN layers and blueshifted by increasing the graphene’s chemical potential. Such flexible control of infrared perfect absorption offers a new tool for controlling electromagnetic waves and has potential applications in photodetection and other light control applications.

## Introduction

In photonics applications such as selective thermal emitters^1^, photovoltaics^2^, polarization splitters^3^, infrared detectors^4^, molecular detection^5^, cloaking^6^, and plasmonic sensors^7^, the underlying mechanism is absorption of incident photons. The only known approach for achieving perfect absorption is to utilize either complex structures^8–13^ or destructive interference mechanism^14,15^. The advent of nanotechnology makes it possible to fabricate two-dimensional materials to control light at nano-scale, which is difficult to achieve using conventional bulky structures^16–19^. In recent studies, two-dimensional materials demonstrated perfect absorption using either surface plasmon resonances as in black phosphorus^20^, or interferenceless mechanism using two-dimensional van der Waals crystal h*exagonal boron nitride* (hBN)^21^. In the composite of graphene and black phosphorus, perfect absorption is reported to be achieved near the vicinity of plasmon resonance of black phosphorus. The limitation of using black phosphorus lies in the fabrication stage where the organic layer is incorporated between the ultrathin layer of graphene and layers of black phosphorus. Though, this composite achieved near-perfect absorption, but in terms of practical realization the additional layer provides additional complexity of understanding the physics behind the hyper-structure^20^. The structure can also exhibit wide-angle robustness, tunability, and near-perfect absorption. In another application using only hBN crystal, by employing impedance matching at the interface of the crystal with the air, the perfect absorption is achieved but for *TM* polarization only. However, the challenge is the difficulty of tuning the structure's angle and frequency due to hBN crystal's rigidity. The composition of hBN and graphene hyper-structure was found to possess polarization splitting of TM and TE waves ^4^. This is achieved by making *TM* polarization opaque and *TE* polarization transparent. It is insensitive to light incident angles, but the hyper-structure is tunable due to inclusion of graphene. However, despite such progresses, finding a crystal that achieves perfect absorption for both *TM* and *TE* polarizations at the same frequency for a wide range of light incident angles will provide additional benefits to the potential applications of perfect absorption.

In this work, we provide simple technique of achieving polarization-insensitive infrared light perfect absorption of both *TM* and *TE* polarizations at the same frequency. The underlying mechanism of achieving perfect absorption of this hyper-structure is by employing zero-reflection points of the hyper-structure. Zero reflections imply perfect absorption since the transmittance through hBN substrate is very small. The applications of such zero reflections are in perfect absorptions, coherent perfect absorptions, plasmonic blackbodies, and Brewster angles^22–25^.

Additionally, most of the works have included graphene to provide tunability to the rigid structure, but including hBN with graphene provide another degree of freedom of attaining both redshift and blueshift without changing the structural parameter. This can be attained due to robustness of the hBN thickness to its bandgap, that means one can increase the thickness of hBN to the tune of 100 nm without changing the bandgap or optical response. Another interesting feature of the crystal is its capability of perfect absorption at a wide range of incidence angles for both TM/TE polarization and broadband frequency range (55 THz to 100 THz and beyond) for TM polarization, which means devices made of such hyper-structures can be physically moved at different angles and different frequencies without affecting its perfect absorption performance. Although there are researches on perfect absorptions, the novelty of our structure is in simplicity of the fabrication process compare with mostly complicated structures that are difficult to fabricate^26–31^.

## Materials and methods

**Figure 1:**
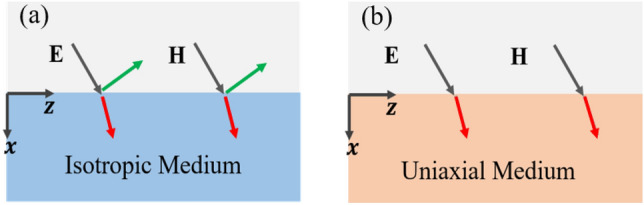
Illustration of light traveling from air into a medium: (**a**) an isotropic medium where transmission, reflection, and absorption happen for both TM- and TE-polarizations; (**b**) a uniaxial medium where perfect absorption is realized for both *TM*- and *TE*- polarizations.

We start with the depiction of electromagnetic wave (*EM*) wave scattering at the interface of air and isotropic medium in Fig. 1a and uniaxial medium in Fig. 1b for both *TM*- and *TE*-waves. It is reported that a uniaxial medium with an optical axis normal to the interface can completely absorb incident *TM*-polarized light at a certain range of angles^32^. The EM wave incident from region 1 with relative permittivity ${\varepsilon }_{1,r}=1$ scatters at the interface, some transmits to region 2 with relative permittivity of either ${\varepsilon }_{2,r}= {\varepsilon }_{1,r}$ as in Fig. 1a or ${\overline{\overline{\varepsilon }}}_{2,r}=({\varepsilon }_{\parallel ,r}, {\varepsilon }_{\parallel ,r},{\varepsilon }_{\perp ,r})$ as in Fig. 1b, while some reflects back to region 1(air). From the classical electromagnetic theory, the reflection coefficient at the interface for *TM*- and *TE*-waves is written as:

1 $${r}_{TM}= \frac{cos{\theta }_{i}- \sqrt{\left({\varepsilon }_{\perp }-{sin}^{2}{\theta }_{i}\right)/{\varepsilon }_{\parallel }{\varepsilon }_{\perp }}}{cos{\theta }_{i}+ \sqrt{\left({\varepsilon }_{\perp }-{sin}^{2}{\theta }_{i}\right)/{\varepsilon }_{\parallel }{\varepsilon }_{\perp }}}$$

and

2 $${r}_{TE}= \frac{cos{\theta }_{i}- \sqrt{({\varepsilon }_{\parallel }-{sin}^{2}{\theta }_{i})}}{cos{\theta }_{i}+ \sqrt{({\varepsilon }_{\parallel }-{sin}^{2}{\theta }_{i})}}$$

where ${\varepsilon }_{\perp }$ and ${\varepsilon }_{\parallel }$ are the permittivity components of normal and parallel to the optical axis of uniaxial medium respectively, while the ${\theta }_{i}$ is the incident angle of the EM wave. From Eqs. (1 and 2) above, for an ideal lossless case for the isotropic case in Fig. 1a, the reflectance is nonzero for all incident angles of *TE* and *TM* waves except for the Brewster angle of *TM* waves^33^. Although loss plays an important role in controlling reflection for both *TE* and *TM* waves, the presence of loss annuls zero reflectance conditions due to impedance mismatch at the boundary of the air and isotropic medium^21^.

For the lossless uniaxial case as in Fig. 1b, there will be a condition where both polarizations achieve zero reflectance $\left|{r}_{TE}\right|=0$ and $\left|{r}_{TM}\right|=0$, i.e., perfect absorption for both polarizations. From Eq. 1 and Eq. 2, the conditions for achieving perfect polarization insensitive absorption for *TM* and *TE* polarizations can be written as

3 $${\theta }_{i} ={cos}^{-1}\left(\sqrt{\frac{({\varepsilon }_{\perp }-1)}{({\varepsilon }_{\perp }{\varepsilon }_{\parallel }-1)}}\right)$$

4 $${\varepsilon }_{\parallel }=1$$

For the lossy case, the above equations combined to achieve the condition by playing around with the complex values of both in-plane (${\varepsilon }_{t} or {\varepsilon }_{||}$) and out-of-plane (${\varepsilon }_{z}or {\varepsilon }_{\perp }$) permittivity of the uniaxial medium. The conditions for IPA can be met when complex in-plane (${\varepsilon }_{t}$) permittivity attains close to unity in Eq. (4), and simultaneously control incident angles $(\mathit{cos}{\theta }_{i})$ of *TM* waves close to the value of the right-hand side of Eq. (3). In a lossy case, the imaginary values of permittivity must combine with real values to satisfy the conditions. This can be observed when the values of effective real permittivity cross each other in the spectrum.

**Figure 2:**
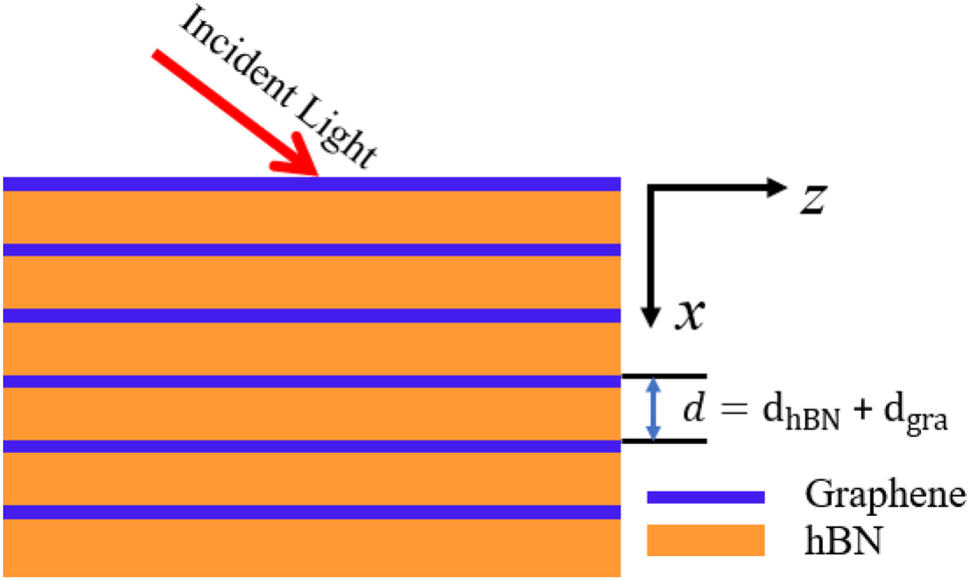
Schematic of the hyper-structure combining graphene and hBN layers.

**Figure 3:**
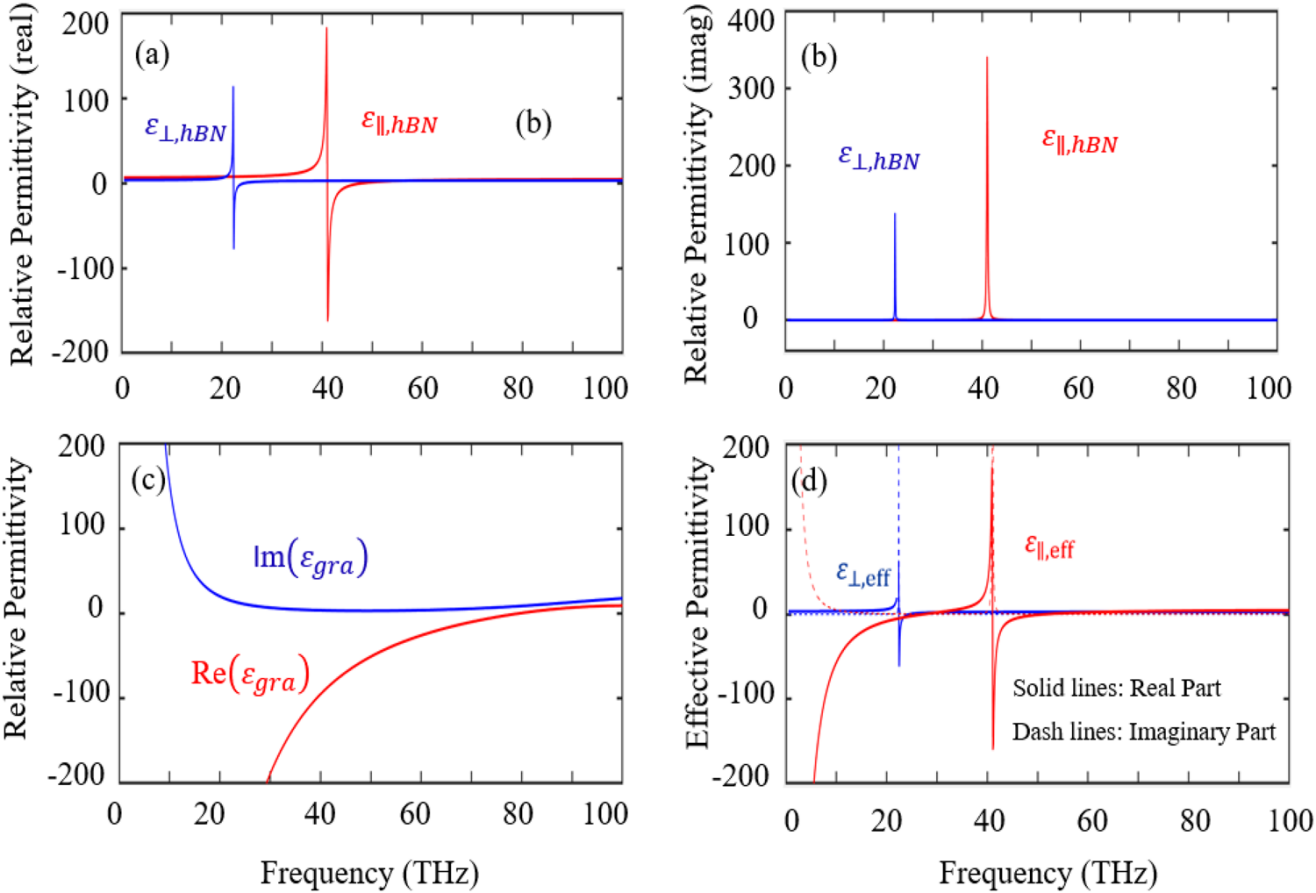
Relative permittivity: (**a**) real part of the permittivity of hBN; (**b**) imaginary part of the permittivity of hBN; (**c**) real and imaginary part of the permittivity of graphene (ε_gra_); (**d**) real and imaginary parts of the effective permittivity of the hyper-structure (ε_eff_).

Next, the proposed hyper-structure as shown in Fig. 2 contains layers of hBN, which is a highly anisotropic material capable of supporting surface phonon polaritons in the upper and lower Reststrahlen bands^34^. Within these bands, the coherent oscillations of the polar lattice result in negative and positive permittivity along the transverse and orthogonal axes of the material respectively. This property makes hBN a strong candidate for the realization of numerous optical phenomena such as hyper-lensing, negative refraction, and strong absorption^35–37^. As compared to two-dimensional materials like black phosphorus or transition metal dichalcogenides, the bandgap of hBN is relatively robust against changes in thickness within the range (5 nm to 100 nm). The bandgap of bulk hBN is around 5.9 eV. The next factor is tunability, most of the works have included graphene to provide tunability to the rigid structure, but including hBN with graphene provide another degree of freedom of attaining both redshift and blueshift without changing the structural parameter. From Fig. 3a, b, we can see the hyperbolic property of the crystal along both (x, y), and (z) axes. The other material in the hyperstructure is graphene, a material that possesses unique qualities compared to other known materials^38^. One of its unique properties is to support surface plasmon polaritons in the terahertz (THz) frequency band^39^. Graphene also has broadband absorption features, ultrafast response to light, and tunable conductivity^40–43^. In the THz frequency band, the real part of the permittivity of graphene $Re({\varepsilon }_{gra})$ is negative with a broadband feature as shown in Fig. 3c. Graphene has a zero bandgap, while hBN has a wide bandgap and their combination supports terahertz frequency. The idea of combining these two important materials is to achieve enhancements and exotic realizations by regulating the electronic transfer of graphene in the hyper-crystal^44^. The viable reactions of the entire hyperstructure can be estimated using the effective medium theory (EMT) when the individual elements (layers) are significantly smaller than the wavelengths range by taking into account the optical characteristics of hBN and graphene mentioned above. The thickness of each period of the multilayer contains an hBN film is denoted as ${d}_{hBN}$ and graphene can also be treated as a thin layer of thickness ${d}_{gra}$; whereas the thickness of the unit cell is denoted by *d*=${d}_{hBN}$ + ${d}_{gra}$ (Fig. 2). The total thickness of the entire hyperstucture is assume to be $200\mu m$. As shown in Fig. 3d, the effective permittivity of the hyper-structure becomes strong hyperbolic which increases the density of states of the free carriers at certain frequencies. The strong density of states is expected at the frequency where the two curves intercept each other. The interception points are where none of the real part is negative, indicating the boundary of the strong density of states of the hyper-structure. The asymmetric nature of the hyper-structure provides a very good condition for enhancing interferenceless perfect absorption^35^. The tunable IPA in the proposed hyperstructure can be achieved either by change changing the chemical potential of graphene or by changing the thickness of hBN.

## Results and discussion

**Figure 4:**
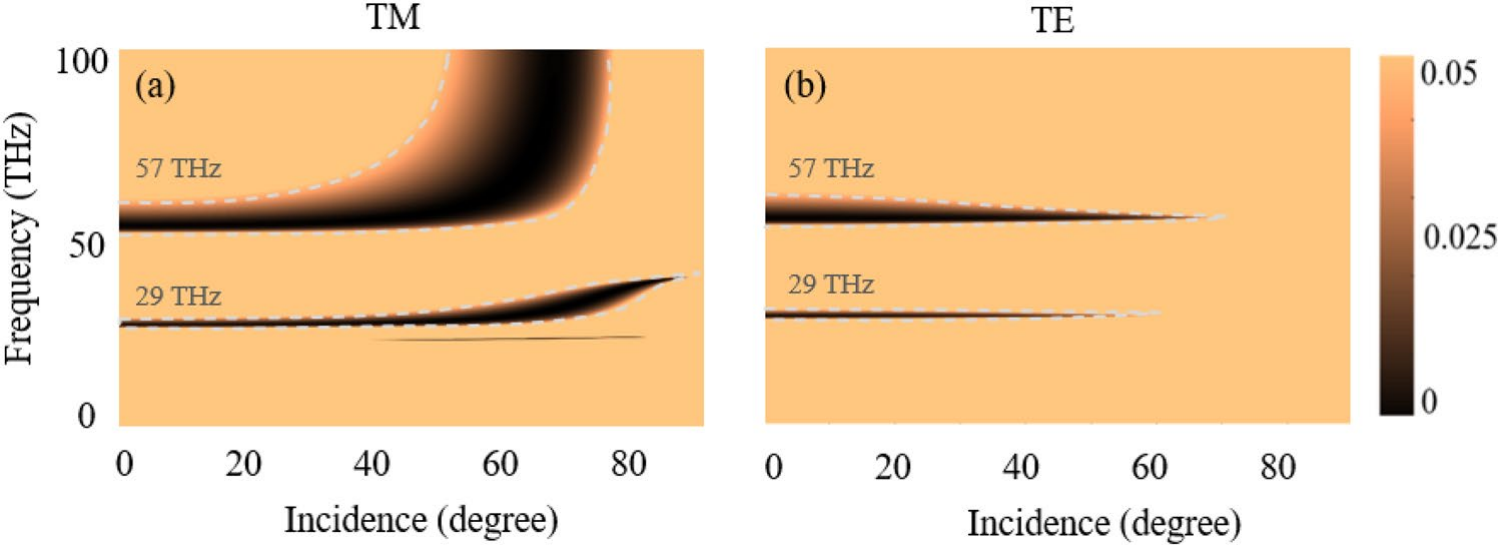
Reflectance spectra: (**a**) TM polarized incident light; (**b**) TE polarized incident light (hBN thickness is 10 nm, graphene thickness 0.34 nm, graphene’s chemical potential 0.2 eV, and temperature 300 K).

Further, the proposed hyper-structure is extremely effective in selectively absorbing terahertz radiation because to the special combination of graphene's remarkable electrical capabilities and hBN's insulating characteristics. Graphene's conductivity may be tuned by external parameters like gate voltage, which enables fine control over its absorption properties. Moreover, hBN's atomically flat and smooth surface reduces scattering losses, resulting in better absorption. The proposed hyperstructures with the following parameters: ${d}_{hBN}=$ 10 nm, ${d}_{gra}$= 0.35 nm ^43^, graphene’s chemical potential of 0.2 eV, and temperature of 300 K, the reflectance spectra of the hyper-structure for both *TE* and *TM* polarized incident light for different incident angles are plotted in Fig. 4. It should be noted that thickness of hBN is more sensitive to the graphene’s chemical potential. The smaller size of the thickness of the hBN exhibit efficient tunabilty effect in the hyperstructr’s and vice versa. Perfect absorption is observed in the region where the reflectance spectra become zero for both *TM* and *TE* polarizations as shown in Fig. 4a, b. It occurs around frequencies of 29 THz and 57 THz (by taking the mid-point of the perfect absorption band) just below the upper and lower Reststrahlen bands. The observed perfect absorption is insensitive to both polarizations and for large incidence angle range (0^0^—55^0^). The reflectance spectra of *TM* polarized light are broader for both frequencies of 29 THz and 57 THz, as shown in Fig. 4a. This is due to presence of ${\varepsilon }_{\perp }$ in the resonant behavior of the hyper-structure. The reflectance spectra of the *TE* polarized light are narrower, as shown in Fig. 4b. This is due to the presence of ${\varepsilon }_{\perp }$ in both scattering of light and in the phonon polariton resonance of the hyper-structure. Moreover, a perfect absorption for a broad frequency band (55–100 THZ) is observed for TM polarization.

**Figure 5:**
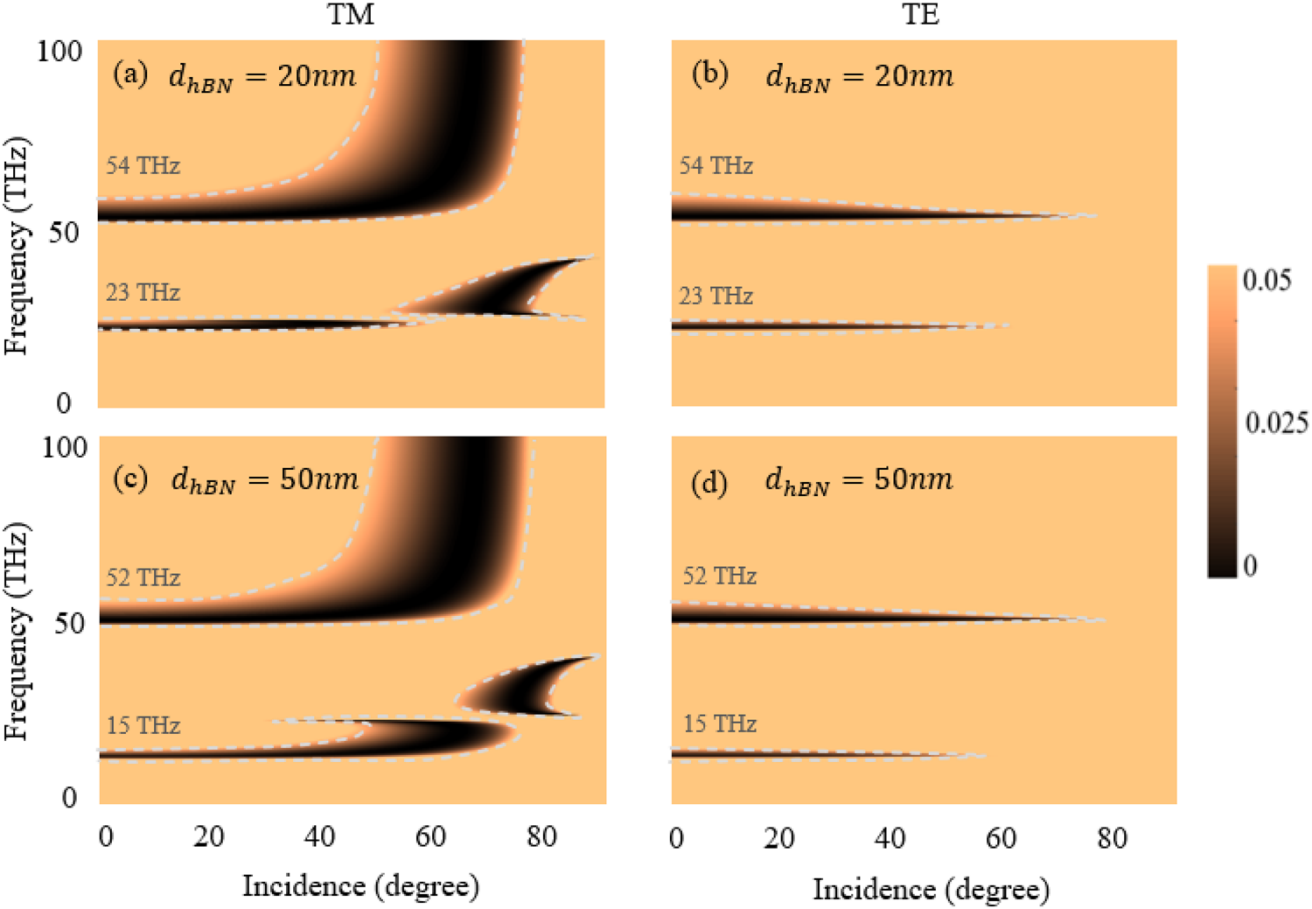
Reflectance spectra for different thickness of hBN: (**a**) TM polarized incident light and hBN thickness of 20 nm; (**b**) TE polarized incident light and hBN thickness of 20 nm; (**c**) TM polarized incident light and hBN thickness of 50 nm; (**d**) TE polarized incident light and hBN thickness of 20 nm (graphene thickness 0.34 nm, graphene’s chemical potential is 0.2 eV, and temperature 300 K).

In order to study the effect of hBN thickness in the hyper-structure on the observed polarization-insensitive perfect absorption, we changed the hBN thickness from 10 to 20 nm and 50 nm. For *TM* polarized light incident on the hyper-structure the perfect absorption is broader and redshifted. For the hBN thickness of 20 nm the reflectance spectrum is redshifted for both Reststrahlen bands to 23 THz and 54 THz, respectively, as in Fig. 5a, while for hBN thickness of 50 nm, it is redshifted further to 15 THz and 52 THz, respectively, as in Fig. 5c. This is due to increase or decrease in the hBN thickness strongly effect the ${\varepsilon }_{||,{\text{eff}}}.$ As compared to Fig. 5a, an increase in the thickness of hBN in Fig. 5c results the intercept points of ${\varepsilon }_{||,{\text{eff}}}$ and ${\varepsilon }_{\perp ,{\text{eff}}}$ to be redshifted in Fig. 3c. Therefore, both the upper and lower Reststrahlen bands are redshifted for about 8 THz and 2 THz, respectively. The broadening of the reflectance spectra in both Fig. 5a and c is due to the domination of the hBN effect in the hyper-structure. This is similar to the nature of the absorption property of single hBN crystal^21^. The hBN and graphene are excellent combinations for hyper-structure as they do not interfere with each other on their respective optical properties^45,46^. This feature helps to control the effect of perfect absorption of the hyper-structure by the virtue of hBN dominance in the THz region. For *TE* polarization in Fig. 5b, d, the reflectance spectrum is redshifted in ways similar to the *TM* polarization in Fig. 5a, c. However, there are differences in the broadening of the reflectance spectrum between hBN thickness of 20 nm and 50 nm. We believe this is due to the dominance peak of the hBN around 20 nm thickness, which reduces as the thickness increases. The *TE* polarized light showed narrower reflectance spectrum compared to the *TM* polarized lights, light with both polarizations undergoes similar redshift when increasing the thickness of hBN in the hyper-structure. It should be noted that increase in the thickness of hBN reduce the tunabilty effect of graphene in the hyper-structure. Also, we avoid varying the thickness of graphene in the hyper-structure due to entirely different optical properties of multilayers of graphene compared to the monolayer^47^.

**Figure 6:**
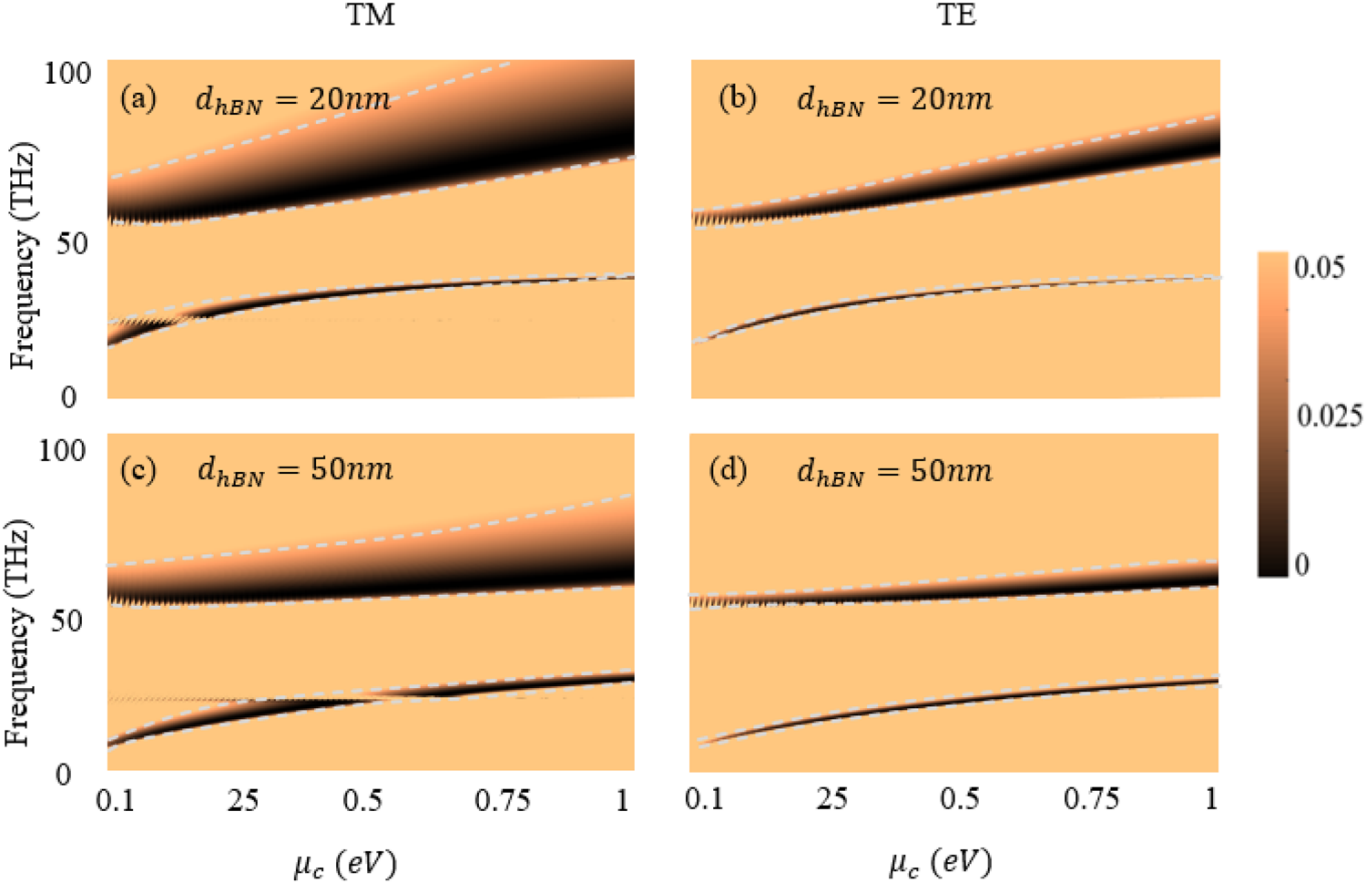
The effect of varying chemical potential ${\upmu }_{{\text{c}}}$ from 0 to 1 eV for: TM-polarized light (**a**) and (**c**) correspond to 20 nm and 50 nm hBN thickness respectively, and TE-polarized light (**b**) and (**d**) correspond to 20 nm and 50 nm hBN thickness respectively.

Moreover, the chemical potential of graphene is an important optical parameter that provides tunability to numerous photonics phenomena^48^. For the proposed hyper-structure, we studied the effect of varying chemical potential of graphene^48^. Figures 6a and c illustrate the effects for *TM* polarized incident light with hBN thickness of 20 nm and 50 nm respectively while chemical potential of graphene varies from 0 to 1 eV. Intuitively, the spectrum of the perfect absorption experiences redshift by increasing the thickness of hBN. However, by increasing the chemical potential the frequency of the perfect absorption is increased too. But the change is more pronounced when hBN thickness is 20 nm (Fig. 6a, b than when it is 50 nm (Fig. 6c, d. This is due to increase in the chemical potential of graphene with small thickness of hBN result in high carrier mobility result in more tunability. There are points of singularities in lower branch of spectrum where perfect absorption is not observed for *TM* polarized cases. Such topological phase singularity in Fig. 6a, c are resulting from crossing of optical response of graphene to the dispersion of zero reflection points of hBN^49^. For the *TE* polarized incident light, the perfect absorption spectrum is narrower compared to the *TM* polarized case. Similar to *TM* polarized case, the frequency of perfect absorption is increasing with increasing chemical potential of graphene, and decreasing by increasing the hBN thickness in the hyper-structure. However, the dramatic difference for *TE* polarized case is in the broadening of the reflectance spectrum when chemical potential is increased, as shown in Fig. 6b, d. These important findings provide means to tune the perfect absorption for redshift and blueshift depending on the needs of different applications.

**Figure 7:**
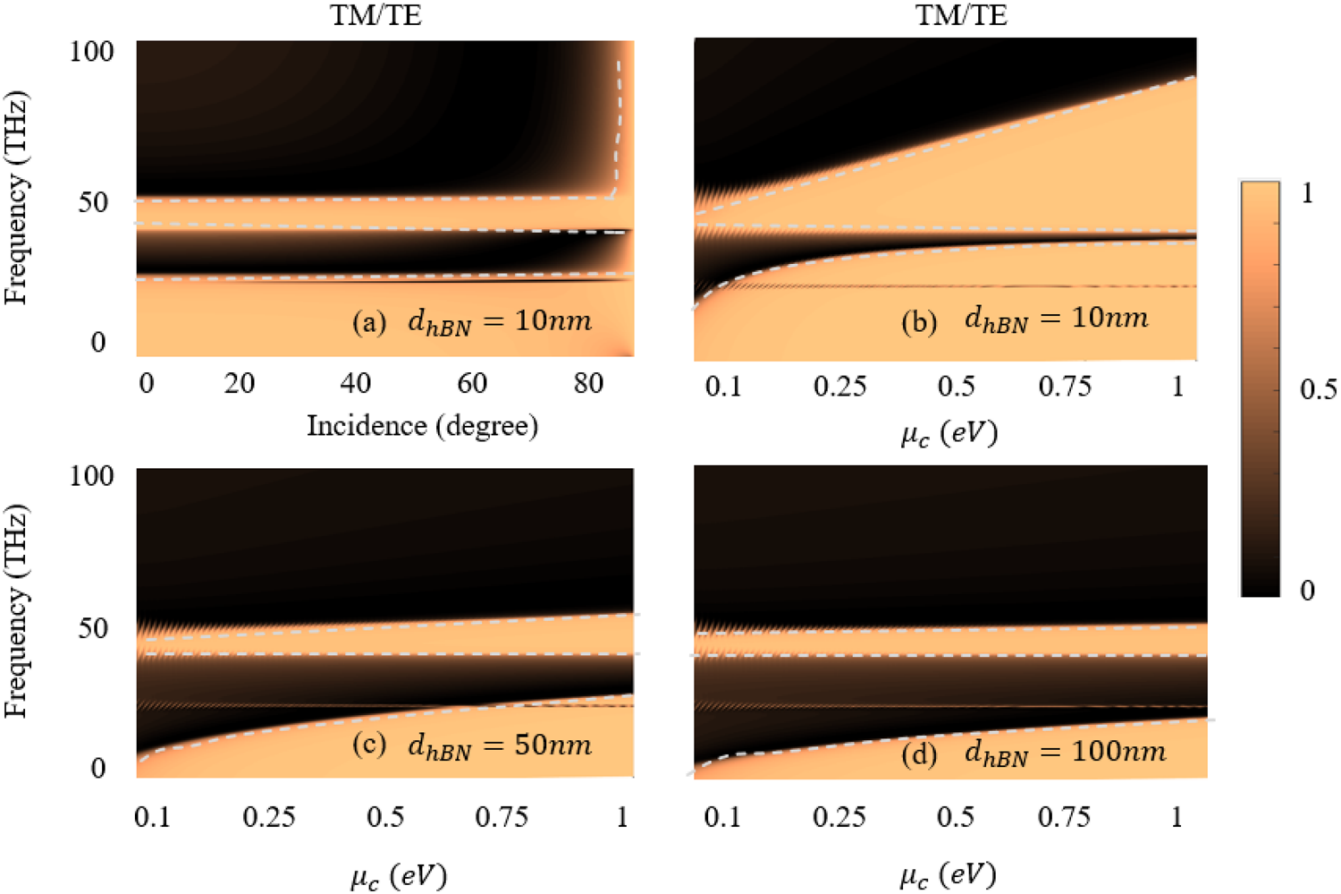
Shows the ratio of TM/TE with reflection and absorption (**a**) when the chemical potential is fixed at 0.2 eV (**b**–**d**) Shows the effect of the tunability for different thicknesses of hBN.

Furthermore, the unique polarization ratio underlines the ability of material’s to efficiently replicate THz waves, making it a promising candidate for applications demanding controlled THz transmission. The interaction between the graphene and hBN layers in the proposed hyperstructure contributes to the variation of polarization, facilitating a desirable balance between reflectance and absorbance at THz frequencies, thereby revealing potential avenues for advanced THz device design and technology. Figure 7 exhibits ratio of *TM/TE* polarization whereas both (*TM* and *TE*) go to one, indicating high reflectance or low absorbance. Figure 7a shows bands of high reflectance and low absorbance at the working frequency 50.58 and 24.4 THz while the chemical potential is fixed at 0.2 eV. Since, the high reflectance band is increases with an increase in the chemical potential. The maximum *TM/TE* reflectance and tunability exhibits in Fig. 7b when the hBN thickness is 10 nm. However, both the *TM/TE* high reflectance and the tunabilty effects are gradually reduces with an increase in the thickness of the hBN shown in Figs. 7b–d.

In practical, graphene-hBN configurations have inspired extensive interest since its creation by a layer-by-layer transfer method^50^. Such hyper-structure as described in many works and has been reported and experimentally demonstrated that how the optical response of the hyper-structure outshines many composites^51–53^. The proposed hyperstructure can also be realized for THz absorption through techniques such as van der Waals epitaxy, chemical vapor deposition, and transfer methods, which enable precise control over layer thickness and stacking configurations^54^. Most recently, the graphene-hBN hyper-structure were obtained with scalable approaches in which hBN continuous films were grown via ionbeam- assisted physical vapor deposition (IBAD) and graphene single-crystal matrixes grown on metal^55^. In such methods, first, continuous films of nanocrystalline hBN with thicknesses of nanometer range can be synthesized on substrate through a physical vapor deposition approach. Then, monolayer graphene single-crystals were transferred with a semi-dry approach on the target IBAD-hBN substrates^56,57^. Finally, different techniques such as terahertz-time domain spectroscopy, Fourier transform infrared spectroscopy and photomixing techniques can be used for the measurements of THz absorption spectrums.

## Conclusion

We analytically and numerically demonstrate that at particular infrared frequencies, a hyper-structure made up of graphene-h*exagonal Boron Nitride (*hBN) is able to nearly perfectly absorb incident light at broadband terahertz frequency range irrespective of its polarizations. The main reason for combining graphene and hBN is to explore their unique optical properties which remain unaffected by the presence of each other. The infrared perfect absorption can be redshifted by increasing the thickness of the hBN layers and blueshifted by increasing the graphene’s chemical potential. Such flexible control of infrared perfect absorption offers a new tool for controlling electromagnetic waves and has potential applications in photodetection, imaging and many other light control applications.

## Data Availability

Data underlying the results presented in this paper are not publicly available at this time but may be obtained from M.I. (imran89@szu.edu.cn) and R.L. (rujiangli@xidian.edu.cn) upon reasonable request.
